# Advances on the Transfer of Lipids by Lipid Transfer Proteins

**DOI:** 10.1016/j.tibs.2017.05.001

**Published:** 2017-07

**Authors:** Louise H. Wong, Alenka Čopič, Tim P. Levine

**Affiliations:** 1 UCL Institute of Ophthalmology, 11-43 Bath Street, London EC1V 9EL, UK; 2 Institut Jacques Monod, CNRS, UMR 7592, Université Paris Diderot, Sorbonne Paris Cité, F‐75013 Paris, France

**Keywords:** lipid exchange, nonvesicular traffic, oxysterol binding protein-related proteins, tubular lipid binding proteins

## Abstract

Transfer of lipid across the cytoplasm is an essential process for intracellular lipid traffic. Lipid transfer proteins (LTPs) are defined by highly controlled *in vitro* experiments. The functional relevance of these is supported by evidence for the same reactions inside cells. Major advances in the LTP field have come from structural bioinformatics identifying new LTPs, and from the development of countercurrent models for LTPs. However, the ultimate aim is to unite *in vitro* and *in vivo* data, and this is where much progress remains to be made. Even where *in vitro* and *in vivo* experiments align, rates of transfer tend not to match. Here we set out some of the advances that might test how LTPs work.

## Trends

LTPs facilitate the essential movement of lipid across aqueous spaces and are defined by *in vitro* experiments.

Recent developments include a novel concept of countercurrent lipid transfer and identification of additional LTP families by bioinformatics.

*In vivo* and *in vitro* data have yet to converge to one complete model.

Advances in *in vitro* characterisation of LTPs provide an opportunity to unite biochemical experimentation to cellular function.

## What Are Lipid Transfer Proteins for?

Along with vesicular traffic between organelles, which is a major subject in membrane cell biology, there are nonvesicular routes for intracellular traffic. Prominent among molecules that traffic by nonvesicular means are lipids that are interchanged between membrane-bound organelles. While lipids are transported as constituents of membrane vesicles, organelles such as mitochondria derive lipids entirely by nonvesicular routes. Pioneering work on nonvesicular lipid traffic focused on the endoplasmic reticulum (ER)–mitochondria route, which has high capacity in both directions [1]. Subsequently, other nonvesicular routes were found, even within the secretory pathway. For instance, lipid traffic between the ER and the plasma membrane is faster than can be accounted by vesicular traffic (half-life, 1–5 min) 2, 3. To solve the riddle of all of this nonvesicular traffic, lipid transfer proteins (LTPs) were postulated as activities that mediate lipid transfer across the cytoplasm. By definition, LTPs stimulate some or all of the following steps: extracting lipid from a membrane, mobilising lipid into the aqueous cytoplasm, and re-inserting lipid into a different membrane.

**Figure 1:**
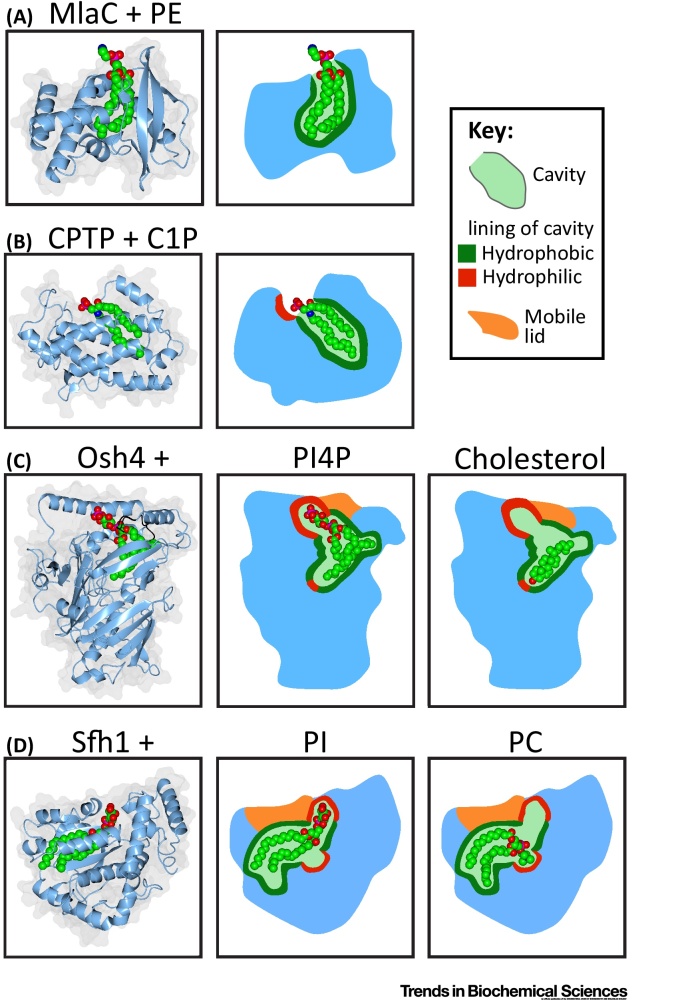
Different Modes by Which Lipid Transfer Proteins Solubilise Membrane Lipids. (A) MlaC (a periplasmic protein) crystallises with phosphatidylethanolamine (PE) but does not interact with its headgroup [6]. (B) Ceramide 1-phosphate (C1P) transfer protein (CPTP) binds the lipid with a hydrophilic patch outside the cavity. (C) Osh4p, a yeast relative of oxysterol binding protein (OSBP), binds phosphatidylinositol-4-phosphate (PI4P) or sterol, with two internal hydrophilic patches. (D) Sfh1, a close homologue of Sec14 in yeast, binds phosphatidylinositol (PI) or phosphatidylcholine (PC), with two internal hydrophilic patches. In both (C) and (D), the lipid is almost entirely shielded from solvent access, but these lipid transfer proteins differ in that for Osh4 (C) there are conformational changes associated with different lipid occupancy, particularly in the mobile lid. By contrast, for Sec14 and homologues including Sfh1 (D), there is no significant external response to internal occupancy. Left-hand panels: ribbon diagrams with background showing space-filling profiles and lipid ligands as space-fill format (coloured by atom: C = green, O = red, N = blue, P = magenta). Other panels: cartoons with lipid binding pockets lined according to key and major ligands: one in (A) and (B); two in (C) and (D) where the ligands shown in the ribbon diagrams are PI4P and PI, respectively. Ribbon diagrams taken from PDB files with accession numbers: 2qgu, 4k8n, 1zhy, 3spw, 3b7n, and 3b7q.

The first LTPs were identified by their ability to recapitulate lipid transfer in cell-free experiments that contained radio-labelled donor membranes and cold acceptor liposomes 1, 4. LTPs show varying degrees of specificity for the lipids they transfer (Table 1). Structural studies showed that many LTPs shield the hydrophobic portions of the lipid, typically in internal cavities that enclose the lipid with a mobile protein segment, similar to a box with a lid (Figure 1). This makes it energetically possible to carry lipids into the cytoplasm. Underlying any specificity an LTP may have for a lipid headgroup is a hydrophilic binding site that may be inside or outside the cavity (Figure 1). By gene duplication large LTP families have arisen, some with widely divergent lipid specificities [5]. Some families have counterparts in prokaryotes (Table 1), which also need LTPs to shuttle lipids between membranes [6].

**Table 1:** Twenty-Three Protein Families Capable of Trafficking Bilayer Lipids

Superfamily^a^	Family	Human^b^	Yeast^b^	Spec^c^	Ligands^d^	First found^e^	Fold^f^	PDB^g^	Refs
FAD/NAD binding		13	2	≥3	PE ?Other PL	cryst. 2010	α pocket	3k7m	[94]
GLTP^m^		4	0	≥3	GSLs	pur. 1982	α	1sx6	95, 96
Insect allergen repeat (nitrile-specifier detox)		[Insect]^h^		≥3	PLs	cryst. 2013	α	4jrb	[97]
nsLTP		[Plant]		≥3	PLs	pur. 1981	α	1afh	98, 99
Saposin		5	0	≥3	GSLs	pur. 1976	α	1m12	50, 100
SCP-2 (also called nsLTP)		5	1	≥3	Sterols PL	pur. 1980	α	1c44	101, 102
Elicitin/cryptogein		[Plant]		≥3	Sterols	cryst. 1996	α⁄β	1beo	[103]
LppX lipid transporter^i^		Mycobacteria		1	Phthiocerol dimycocerosate lipids	cryst. 2006	α⁄β	2byo	[104]
NPC1 (amino terminus)		2	1	1	Sterols	pur. 2008	α⁄β	3gki	[105]
NTF2^i^, ^j^		Prokaryote^k^		≥3	PLs	cryst. 2009	α⁄β	2qgu	[6]
ORP		12	7	2+^l^	PI4P sterol PS	pur. 1989	β barrel + α helices	1zhy	106, 107
Sec14 (CRAL/TRIO)		28	9	2+	PI PC sterol non-BL	pur. 1976	α⁄β	1aua	108, 109
StARkin	StART	15	0	1+	PL sterols non-BL	pur. 1994	β-grip + α helices	1em2	110, 111
	PITP	5	0	2+	PI PC PA	pur. 1974		1kcm	4, 112
	Bet v I	[Plant]		1	Q3OS	cryst. 2000		1fm4	[113]
	PRELI^TRIAP^	4	3	1	PA PS	hom. 2012		4xzs	19, 20, 22
	LAM	3	6	1	sterols	hom. 2014		None	[23]
TULIP	BPI/Takeout	[14]	0	1+	sterol esters PLs TAG non-BL	pur. 1978/1974	elongated β-grip + α helices	2obd	12, 114, 115
	SMP	5	7	≥3	PLs	hom. 2010		4p42	9, 13
Lipocalin^i^, ^j^		Prokaryote^k^		1	PLs non-BL	pur. 1968	β barrel	3e3c	[116]
LPS transport (LptACD)^i^		Prokaryote		1	LPS	gen. 2008	β jellyroll	2r19	[117]
NPC2/GM2AP		4	1	≥3	Sterols GM2	pur. 1979	β sandwich	1nep/1pub	118, 119, 120
WIF-1		1	0	1+	PC	cryst. 2010	β sandwich	2ygn	[121]

a Superfamilies include two families or more that share structure but not sequence as determined by conventional searches 9, 51. Abbreviations are presented in ^m^.

b Number of genes coding for intracellular lipid transfer proteins in humans and budding yeast (*Saccharomyces cerevisiae*).

c Number of lipid species, as identified by differing headgroups or conjugated groups, bound by a single typical LTP in this family.

d Lipid ligands identified within the entire LTP family.

e How and when the family was first found: purified (pur.), crystallised (cryst,), predicted by remote homology (hom.), or by genetics (gen.); dates of publication were between 1968 and 2014.

f LTP families are classified according to the Structural Classification of Proteins system [87].

g Accession code for the earliest solved structure (where available).

h Square brackets indicate extracellular proteins.

i LTPs in this family are confined to prokaryotes.

j Proteins with this fold vary in overall size, and only some are large enough to bind bilayer lipids.

k None of the many metazoal lipocalin domain containing proteins have cavities large enough for bilayer lipids.

l ‘+’ indicates that detailed studies show that some LTPs in this family bind additional lipids, significance as yet unknown. This may turn out to be a general feature of any LTP studied in detail.

m Abbreviations: CRAL, cellular retinaldehyde binding protein; GLTP, glycolipid transfer protein; GM2AP, ganglioside GM2 activator protein; GSL, glycosphingolipid; LPS, lipopolysaccharide; non-BL, nonbilayer lipid; NPC, Niemann–Pick type C proteins; nsLTP, nonspecific LTP (same name applies to a family of plant LTPs and a wide-spread family that is also called SCP-2 for sterol carrier protein-2); NTF2, nuclear transport factor-2; PL, phospholipid; Q3OS, quercetin-3-*O*-sophoroside; TAG, triacylglycerol; TRIO, triple functional domain protein; WIF, Wnt inhibitory factor.

**Figure 2:**
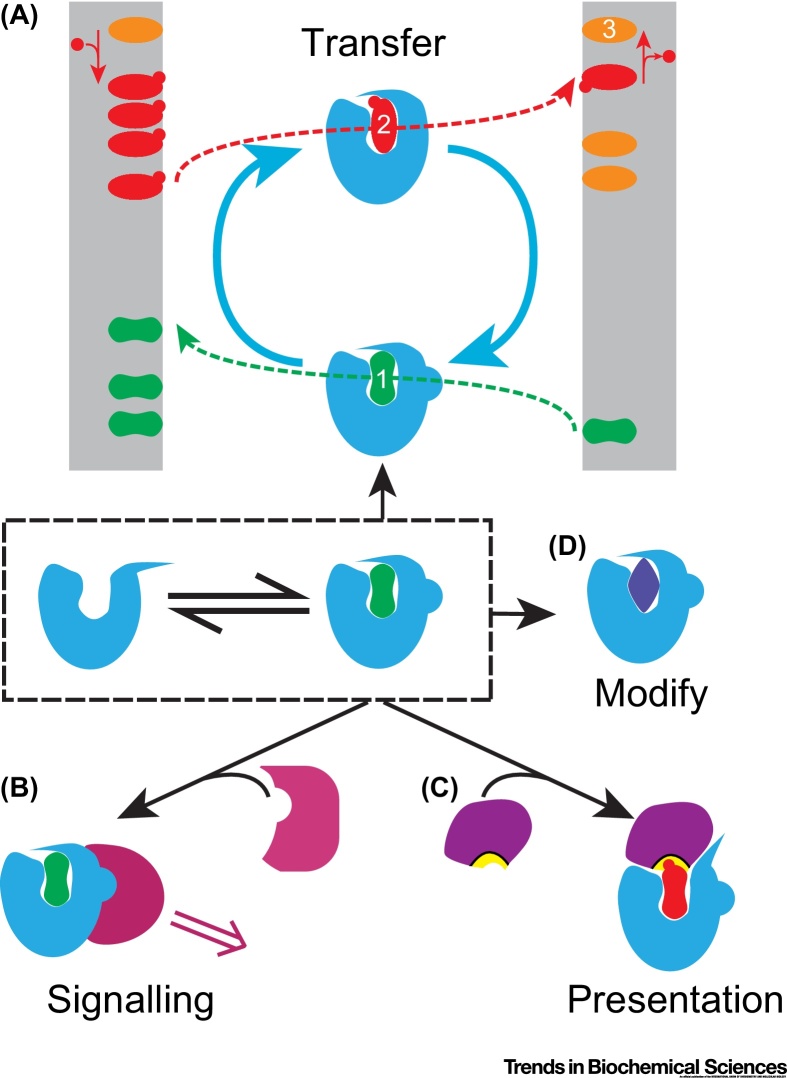
Schematic Illustrations of the Various Functions of a Lipid Binding Domain. (A) Lipid traffic by lipid transfer proteins (LTPs). Either transfer or exchange of lipid can take place at one membrane. Here a countercurrent model is shown, where one LTP (blue) exchanges two lipids (numbered 1 and 2, shown in green and red, respectively) between two membranes. In this example, a steep gradient of Lipid #2 is maintained by its synthesis from Lipid #3 (orange) on the left side, and conversion back to Lipid #3 on the right side. Such a gradient can drive the counterexchange of Lipid #1 up a gradient, albeit this gradient is less steep than that of Lipid #2. This has been shown for oxysterol binding protein (OSBP) homologues, where Lipid #2 is phosphatidylinositol-4-phosphate, Lipid #3 is phosphatidylinositol, and Lipid #1 can be either sterol or phosphatidylserine [38]. (B) LTP as a sensor: An LTP directly senses a lipid if it changes conformation upon binding a lipid and passes that information to a binding partner. Here an interaction is shown between a signalling protein and lipid-bound LTP, whereas the non-lipid-bound form does not interact. This might lead to lipid-dependent signalling, as has been shown for OSBP [48]. Lipid-dependent conformations could also be important for lipid transfer reactions, for example, in membrane targeting (not shown). (C) LTP as a presenter: the LTP–lipid interaction exposes part of the lipid (typically the headgroup) to other proteins, for example, an enzyme (purple) that modifies the lipid (turns from green to red). This applies to the presentation of glycosphingolipids to hydrolytic enzymes by GM2 activator protein (GM2AP) [49]. LTPs that enclose lipid inside a cavity, such as Sec14, may still present lipids as they enter or leave the cavity (not shown) [43]. (D) LTP with an additional lipid modifying function: a protein that can solubilise a lipid ligand will thereby have properties of an LTP; the same protein may also act as a lipid modifying enzyme with other substrates, as is the case for GM2AP when it interacts with phosphatidylcholine and platelet activating factor [52].

The search for accelerators of lipid traffic has led to the identification of LTPs that meet the *in vitro* definition of recapitulating the lipid transfer activity. However, by solubilising lipids an LTP can act equally well as a lipid sensing protein or as a lipid presenting protein, which would lead to signalling or lipid modification (Figure 2) [7]. Mechanistic understanding is required to distinguish between these possibilities, though they may not be mutually exclusive and may also depend on the physiological state of a cell. This article reviews recent advances and describes how future progress might be made using *in vivo* and *in vitro* experimental approaches that test the role of LTPs in net lipid transfer.

## Redrawing the Picture of Lipid Traffic

The LTP field has been reinvigorated this decade by a few major developments. Here we describe in detail two recent advances: an increase in the number of known LTPs, particularly at membrane contact sites; and an unexpected finding that oxysterol binding protein (OSBP)-related proteins (ORPs) solubilise phosphatidylinositol (PI) 4-phosphate (PI4P), which provides a way to generate lipid gradients.

### LTP Numbers Have Risen Dramatically since 2010, Especially at Contact Sites

The number of LTPs that are known to exist has increased partly as a result of predictions using structural bioinformatics tools such as HHsearch, which detects remote homology with great sensitivity [8]. The first example is the discovery that so-called synaptotagmin, mitochondrial and lipid binding protein (SMP) domains of previously unknown function are intracellular homologues of extracellular tubular lipid binding proteins (TULIPs) [9]. These are known to traffic lipids [10], and contain dimeric LTP domains that can form a long hydrophobic tunnel 11, 12. Bioinformatic approaches predicted SMP domains as intracellular TULIPs, and thereafter crystal structures were obtained for the SMP domains of extended-synaptotagmin-2 (E-Syt2) [13] and Mdm12 [14]. Like extracellular TULIPs, SMP domains dimerise to form a long hydrophobic tube that binds up to four lipids (two per monomer). The TULIP domain of E-Syt2 binds glycerolipids preferentially to sterol by a factor of up to 5:1 13, 15. While E-Syt2 shows little or no headgroup specificity, Mdm12 shows considerable specificity for cationic headgroups [16], possibly because of binding to the acidic side chain D255, found only in Mdm12 [14].

Remote structural homologies have also added two large LTP families to the StARkin superfamily, which includes proteins related to the steroidogenic acute response protein (StAR) and homologues of the major allergen from birch *Betula verrucosa* (Bet v I) (Table 1) [17]. The first new StARkin family contains the PRELI proteins (proteins of relevant evolutionary and lymphoid interest; in yeast called Ups for ‘unprocessed Mgm1’). Unlike intracellular TULIP proteins, PRELI/Ups proteins had been previously implicated in lipid metabolism [18]. Their predicted role as LTPs [19] has since been strengthened by PRELI/Ups crystal structures 20, 21, 22. These show PRELI/Ups in complex with a small accessory subunit TP53-regulated inhibitor of apoptosis-1, (TRIAP1; Mdm35 in yeast), which adds extra helices on to the StARkin β‐grip domain, structurally similar to an extra helix found in PI/phosphatidylcholine (PC) transfer protein (PITP) members of the StARkin superfamily (Table 1). The other new family of StARkin proteins contains the LTPs anchored at membrane contact sites (LAMs), a group of proteins with no prior link to lipid metabolism [23]. In this family, the prediction of a StARkin structure is supported by findings that they transfer sterol *in vitro* [24].

Virtually all intracellular TULIPs and LAMs studied so far are found at contact sites [25]. These are narrow (10–30 nm) gaps between organelles that can be spanned by individual proteins. Contact sites have been found between an increasingly wide range of organelles 25, 26, 27. They were already known to contain a range of LTPs, in particular and many OSBP-related proteins (ORPs, in yeast also called OSBP homologues, Osh), ceramide transfer protein (CERT), PITPs related to RdgB in flies (Nir in humans) [28], and possibly the glycolipid transfer protein four-phosphate adaptor protein-2 (also called FAPP2) [29]. These LTPs use similar combinations of domains to target contacts between the ER and organelles of the exocytic and endocytic pathways. The intracellular TULIP and LAM families all localise to contact sites in a different way, as they are irreversibly embedded in the ER through transmembrane domains.

In addition to these predictions from remote homology, during the same period (2010 to present day) three unexpected LTP families were discovered through structural work that revealed proteins with cavities containing bilayer lipids (Table 1). Thus, structural approaches overall have increased LTP numbers considerably (80 → 106 in humans, 18 → 37 in yeast).

### Two Lipids Drive Faster Than One

New mechanistic understanding of lipid traffic has grown from a detailed analysis of anomalies in lipid traffic by ORPs: PI4P (and to a lesser extent PI4,5P_2_) was known to strongly influence ORP-mediated lipid transfer, but the mechanism was unclear [30]. Work from the labs of Guillaume Drin and Bruno Antonny resolved this by showing that Osh4 not only binds the headgroups of PIPs via basic residues on its surface but can also transfer PI4P [31]. For the transfer activity, PI4P binds inside the hydrophobic cavity at a site that overlaps the previously characterised internal sterol binding site (Figure 1C) [31]. The PI4P binding site is the most conserved part of ORPs, thus PI4P is likely to be the common ligand among them. This finding and its interpretation have transformed the field because they have led to a model where ORPs at ER contact sites can drive forward the traffic of one lipid in a countercurrent that is powered by PI4P.

One of the key facts in the countercurrent model is that PI4P is synthesised by PI 4-kinases (3 in yeast, 4 in humans) in the cytofacial leaflet of the late Golgi, plasma membrane, or endosomes, and that PI4P is destroyed in the ER by Sac1. The implication of the internal binding site for PI4P is that any ORP can transfer it from a site of synthesis to the ER. Because PI4P is hydrolysed at this site, the same ORP is unlikely to pick up PI4P for traffic out of the ER. Instead, it will take another lipid. The second specificity for ORPs varies: for OSBP and Osh4 it is sterol, for ORP5/8 and Osh6/7 it is phosphatidylserine (PS) 32, 33, 34, and it is not known in other cases (e.g., Osh3 [35]). Thus, the asymmetric distribution of PI 4-kinase and PI 4-phosphatase can create multiple lipid gradients (Figure 2A) 36, 37. In this way, ORPs resemble ion antiporters carrying out secondary active transport of one ion up a gradient by harnessing the energy created by another ion flowing down a steeper gradient. Among the strongest *in vivo* evidence that supports the countercurrent model is the recruitment of PI 4-kinases and ORPs to viral replication sites that become highly enriched in both PI4P and cholesterol [38]. Lipid countercurrents are likely to be highly efficient at membrane contact sites, as shown by the rapid delivery of PS from ER to plasma membrane in yeast [34]. However, contacts are not mechanistically essential for countercurrent 31, 37.

Asymmetries in lipid distribution are seen throughout the cell 39, 40. The countercurrent model of ORP function is very appealing because it explains how some of the asymmetries in lipid distribution might be achieved. Nevertheless, more detailed studies are required to determine whether the production rate of PIPs can match the required lipid transfer rate. The model has raised the importance of considering second lipids for LTPs other than ORPs [41]. Other LTPs with second lipids that might engage in countercurrent include alpha-tocopherol transfer protein among other Sec14 homologues 42, 43 and RdgB/Nir 44, 45, where PI is likely to be the lipid under metabolic control.

The focus on countercurrents prompts a greater interest in minor ligands for all other LTPs, especially if they could engage in a countercurrent. For example, CERT mediates the transfer of ceramide from the ER to the trans-Golgi network (TGN). After delivery, ceramide is converted to sphingomyelin with co-production of DAG, which is potentially toxic to the TGN. The minor ability of CERT to solubilise and transfer DAG [46] may therefore be physiologically relevant, and ceramide plus DAG may engage in a countercurrent mediated by CERT to extract DAG from the TGN, and to deliver ceramide.

## Nonlipid Transfer Functions for LTPs

In addition to transferring lipids, LTPs can also act as lipid sensors and lipid presenters. For example, some StARkin domains are found in transcription factors, where their ability to bind lipid correlates with transcription [47]. For LTPs to act as lipid sensors, LTP–lipid interactions must produce unique structural conformations (Figure 1A–C) that then cause signalling events (Figure 2B). To establish a function in lipid sensing, it is important to exclude lipid transfer as the cause of the LTP-derived signal. The easiest way to separate the two activities might be to show that the main function of an LTP is (or is not) lipid transfer, for example, by heterologous replacement (see below).

LTPs may also function as lipid presenting proteins (also called lipid chaperones [5]) when they present part of a lipid, typically the hydrophilic head group, to another protein (Figure 2C). For example, GM2 activator protein (GM2AP) presents glycolipids such as GM2 to a hydrolysing enzyme HexA, acting as an essential enzyme cofactor [48]. Some presenting proteins such as saposins are LTPs, but they are also called ‘liftases’, because they lift lipids out of the bilayer, enhancing access to them by enzymes [50]. Presentation of lipids by LTPs such as Sec14 and its homologues, where bound lipid is located entirely within an internal cavity (Figure 1D), has been proposed to occur during lipid entry into and exit out of the binding pocket, in which case the act of lipid exchange is key [43]. In addition, lipid presentation may occur between cells, for example, by the CD1 major histocompatibility complex, which presents lipid headgroups to T-cell receptors [51].

Finally, LTPs can have lipid-modifying functions (Figure 2D). For example, GM2AP not only presents the sphingolipid GM2, but also has a hydrolase activity for glycerolipids that have choline headgroups, including PC and platelet activating factor (PAF) [52], although the physiological relevance of this activity is not yet clear. There are other examples where structural and sequence homologues of LTPs are known as lipid-modifying enzymes 53, 54, and it is appealing to speculate that LTPs originated from lipid-modifying enzymes, as has been observed for other enzyme–non-enzyme pairs [55].

## Approaches to Study Lipid Transfer by LTPs

There are many different experimental approaches to understanding LTPs, with an obvious split being between *in vitro* and *in vivo* experiments. Each approach asks a specific set of questions about a lipid transport step, which provides useful information that other approaches may not probe.

### Cell-Free Reconstitution of Lipid Traffic

**Figure 3:**
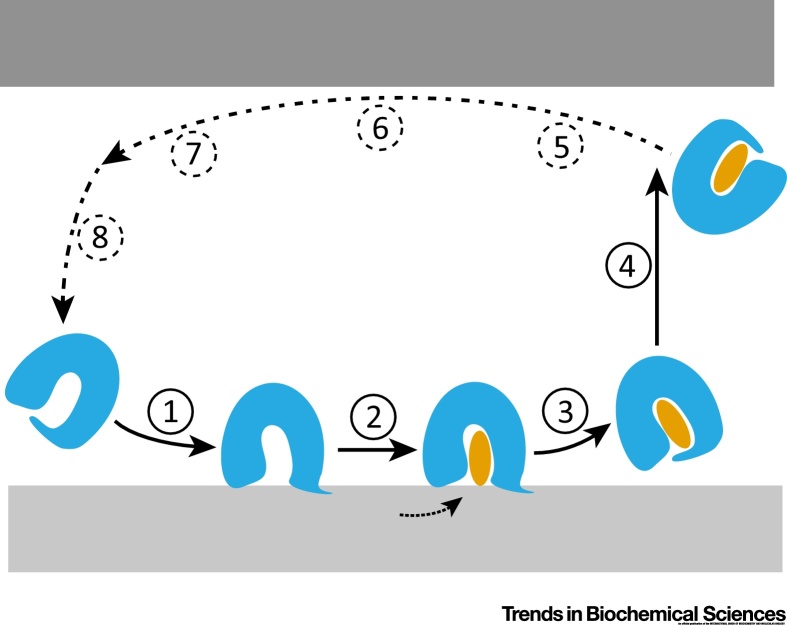
The Minimal Number of Steps in a Lipid Transfer Reaction. The overall lipid transfer reaction can be dissected into eight substeps. 1: lipid transfer protein (LTP) binds to donor membrane. This step may be regulated by lipid occupancy and membrane composition to reduce irrelevant membrane interactions. 2: Lipid extraction; or, if the LTP works as a lipid exchanger, swapping of lipids. 3: LTP–lipid complex dissociates away from the membrane. The occupancy of the pocket can also influence release of the LTP. 4: Diffusion between membranes, until the LTP encounters the acceptor membrane. Steps 5–8 are essentially as 1–4, but at the acceptor site. Steps 1–3 and 5–7 should involve conformational changes (e.g., the closing of a lid after membrane dissociation of an LTP–lipid complex), which could make reverse reactions unfavourable. Additional intermediates (protein conformations) are also possible.

Even the simplest LTP activity will consist of many discrete steps (Figure 3). To reconstitute such a reaction, we need purified LTP, knowledge about the membranes between which it operates to mimic them with liposomes, and various readouts to follow the reaction and possibly the individual steps in real time. In such a reductionist approach, protein activities and individual parameters can be tested directly one by one. Furthermore, we can gain information on the duration of reaction steps, the number of protein conformations, and the energy of the reaction.

An *in vitro* system must adequately approximate the *in vivo* reaction, which may require many components and so seems daunting. However, highly complex reactions such as the initiation of DNA replication [56] or vesicle fusion [57] can be reconstituted in cell-free systems that are highly informative (Box 1). In contrast to these processes, lipid transfer reactions can be observed with single proteins 4, 21. More complex reconstitutions have now been carried out with contact site bridging by membrane receptors [58], countercurrent generation by PI 4-phosphatase [37], and Ca^2+^-induced lipid transport 15, 59. For lipid countercurrents, it is clear that LTPs such as ORPs preferentially unload their first ligand when the second ligand is available in the acceptor membrane [31].

Box 1 **In Vitro Reconstitution Approaches: Lessons from SNARE-Dependent Membrane Fusion**

The complexity of a living cell can hardly be matched by an *in vitro* reconstitution. However, a continuous effort at improving a biochemical reaction in a membrane environment can yield enormous insights, as can be shown by the example of soluble NSF attachment protein (SNAP) receptor (SNARE) proteins and membrane fusion. One enduring argument against the importance of SNAREs in membrane fusion was that the fusion reaction with liposomes and purified SNARE proteins was at first very slow [57]. It was difficult to imagine how such a reaction could drive the release of neurotransmitters in the brain (five orders of magnitude faster!). It was only through the efforts and persistence of many researchers over many years that the importance of SNAREs in membrane fusion has become widely accepted [85]. Several lessons from this venture may be informative for studies of LTPs. Crucial advances were made by (i) identifying core and accessory factors, (ii) defining rate-limiting steps, and (iii) understanding the impact of membrane composition, in this case, the lipids. When one compares the initial reconstitution experiments with the most recent ones, the core machinery remains the same: a set of SNARE proteins distributed between donor and acceptor membranes. However, including additional proteins and lipids to better mimic the composition of authentic organelles and understanding several ‘off-pathway’ reactions have together combined to tremendously increase efficiency, speed, and fidelity of the reconstituted reaction 86, 87.

The recent increase in interest in lipid transport at membrane contact sites has led to the identification of many new players and many new hypotheses, but also, not surprisingly, to controversies and impatience to have ‘the real answer’. In the case of SNARE-dependent fusion, *in vitro* reconstitution assays at first only qualitatively approached what happens *in vivo*. However, this strategy enabled gradual progress to a point where the reaction in the tube accurately represents the reaction in the cell, along the way clarifying the contributions of individual players.

Alt-text: Box 1

Replicating the membrane environment is a difficult issue, accounting for many ways in which *in vitro* LTP assays might give false-negative or inefficient results. For example, the donor or acceptor composition may produce LTP–membrane interactions that are either too weak for LTP association or too strong for LTP dissociation [19]. Another factor is that net transfer between liposomes *in vitro* will alter lateral pressures in the liposomes’ external leaflets unless a component of the liposomes can flip to rebalance leaflet contents. Since flipping is slow for glycerolipids, assays *in vitro* might under-report LTP activity. However, there are ways around this problem by designing assays where the LTP returns a lipid from acceptor to donor [21]. Maybe less obviously, an LTP reaction can also give false positives. If the membrane composition or the curvature leads to an unstable bilayer, natural barriers to lipid extraction might be overcome. Reconstitutions can mimic the relevant organelles by using representative liposomes in terms of polar headgroups, acyl chains, and membrane curvature to address topics such as the influence of bilayer packing defects on lipid transfer [37].

Other properties of cellular membranes are extremely difficult to replicate in a synthetic system. These include transverse and lateral heterogeneity and the large number of other proteins, which leads to crowding 60, 61. Among the elementary steps of the LTP cycle reconstituted in cell-free reactions, those that are more likely to be affected by experimental approximations are protein diffusion, and every step that corresponds to an ‘on’ reaction, such as collisions of the protein with the membranes (Figure 3; Steps 1, 4, 5, and 8). By contrast, ‘off’ reactions should be less affected by the *in vitro* approximation because they correspond to dissociation between well-defined components. Fast reactions require stopped-flow kinetic measurements, but some steps, such as membrane binding and lipid extraction, are difficult to uncouple experimentally. Limited parts of the LTP reaction cycle can be simulated using molecular dynamics 34, 37.

Other important ingredients that might be missing from current assays are cytosolic or membrane proteins that reduce the energy barrier to lipid extraction, which is the rate-limiting step [62]. Some accessory domains that accompany LTPs may alter the stability of the bilayer locally 63, 64. Some interactions might orient LTPs to enhance their productive interactions with the membrane. In addition, at high local concentrations, which are found at membrane contact sites, LTPs and associated domains may show co-operativity. Finally, there are regulatory interactions that might provide energy inputs that accelerate rates of productive lipid transfer by LTPs. Phosphorylation–dephosphorylation cycles are known to regulate LTPs [65]. In addition, some LTPs interact with chaperones; for example, Osh1 with the AAA-ATPase Afg2p [66]. The roles of any of these factors have not yet been tested *in vitro*.

### Some Lipid Traffic Pathways Are More Amenable to Study Than Others

**Figure 4:**
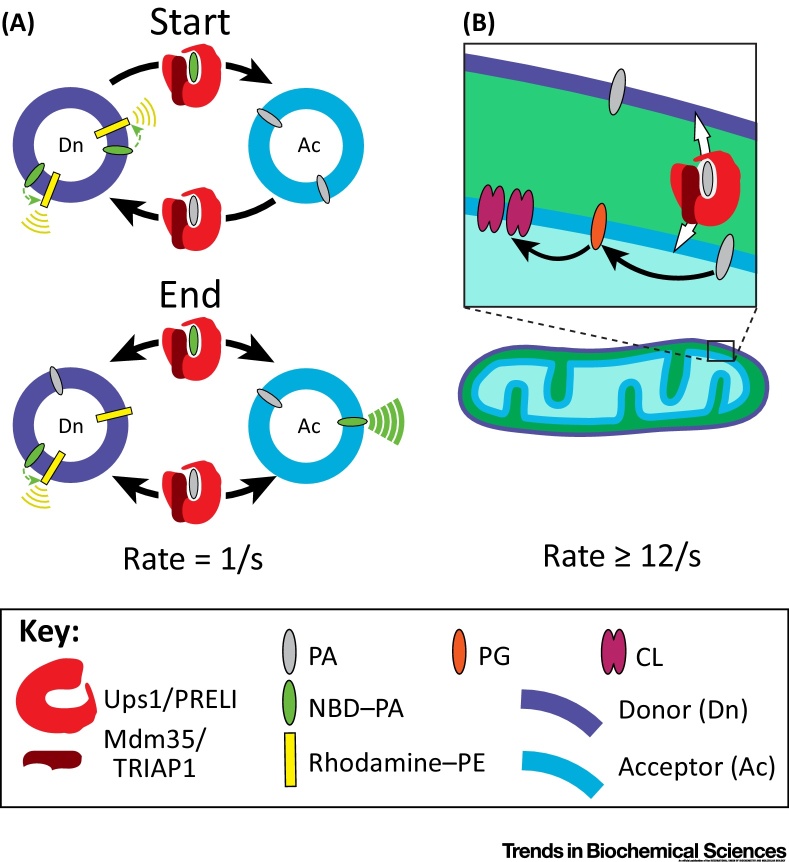
Discrepancies between Rates of Lipid Transfer Protein (LTP) Transfer *In Vitro* and *In Vivo*. (A) *In vitro* rates of phosphatidic acid (PA) transfer by Ups1/Mdm35. In highly reproducible, well-controlled assays by Watanabe *et al.*[21], the calculated rate at which the LTP moves PA from any one liposome to another = 1/s (details in Box 2A). This is the upper limit for net traffic. At ‘Start’, the measured emission from 7-nitro-2-1,3-benzoxadiazol-4-yl (NBD)–PA is inhibited as it undergoes Förster resonance energy transfer (FRET) with rhodamine–phosphatidylethanolamine (PE), which emits at 585 nm (yellow signal). Note that although NBD–PA and rhodamine–PE are diffusing freely in the donor membrane, they are illustrated close together to indicate the proximity for FRET, which is ≤2 nm. As the reaction progresses to ‘End’, the measured NBD fluorescence increases as NBD–PA is moved to acceptor liposomes lacking rhodamine–PE, where it emits at 535 nm (green signal). (B) Estimate of *in vivo* rate of PA import into mitochondria in budding yeast. Cardiolipin (CL) is made in the mitochondrial matrix from a phosphatidylglycerol (PG) and a cytidine diphosphate diacylglycerol, each of which is made from one imported PA molecule. The lower limit of *in vivo* PA transfer by Ups1 is estimated to be 12/s (details in Box 2B). This is >12-fold faster than the rate measured in (A). See Box 2 for potential sources of error. Abbreviations: Ac, acceptor; Dn, donor; PRELI, proteins of relevant evolutionary and lymphoid interest (in yeast called Ups for ‘unprocessed Mgm1’); TRIAP1, TP53-regulated inhibitor of apoptosis-1 (Mdm35 in yeast).

When it comes to studying lipid traffic in living cells, some pathways are more difficult to work on than others. The combination of *in vitro* specificity and *in vivo* localisation of an LTP may suggest its *in vivo* activity. However, the key evidence is whether measured rates of *in vivo* lipid traffic are affected by changes in LTP levels. Such evidence has been obtained in some examples: (i) traffic by CERT of ceramide from the ER to the TGN for sphingomyelin synthesis; (ii) uptake by StAR of cholesterol into mitochondria for steroid hormone synthesis; and (iii) delivery by Ups1p/Mdm35p of phosphatidic acid (PA) from the outer mitochondrial membrane to the inner mitochondrial membrane for cardiolipin synthesis (see Figure 4 and Box 2). In these cases, it has been uncontroversial to suggest that these LTPs mediate net lipid traffic 19, 46, 67. In other cases, it has been far harder to determine the *in vivo* LTP because important features that allow the crucial *in vivo* experiments to work are missing. One of these features is linearity of the transport pathway. For lipid traffic, connectivity of organelles via membrane contact sites is often circular, compared with the more linear secretory pathway, at least in its early stages.

Box 2 **Detailed Analysis of PA Import into Mitochondria by Ups1 in Yeast**

**(A) Evaluation of *In Vitro* PA Transfer Assays by Ups1/Mdm35 (Yeast Homologs of PRELI/TRIAP1) in Watanabe *et al.*** [21].

Analysis of three factors yields a rate of 1/s for the *in vitro* transfer of PA by Ups1/Mdm35 complexesthat is shown in diagrammatic form in Figure 4A: (i) the proportion of transfers measured by fluorescence of NBD–PA, (ii) the rate of signal increase, and (iii) the number of NBD–PA transfers at equilibrium.

(i) Donor (Dn) and acceptor (Ac) liposomes (ratio 1:4, both 100 nm diameter) have initial composition differences: Ac contains unlabelled PA (∼1250 nM in external leaflet). The PA in Dn is replaced by fluorescent NBD–PA (∼250 nM in external leaflet), which is quenched by rhodamine–PE present only in Dn. When LTP is added to both Dn and Ac, there is an increase in NBD fluorescence with first-order kinetics, indicating net traffic of NBD–PA from Dn to Ac (Figure 5B in [21]). We assume equivalent net traffic of PA from Ac to Dn, and that the reaction tends to an equilibrium where the total PA content of each liposome is composed of a 1:5 ratio of NBD–PA and unlabelled PA. Among the distinct movements of the LTP, transfer from Dn → Ac is only one; others will be Dn → Dn, Ac → Ac, and Ac → Dn. Assuming that the LTP interacts equally with Dn and Ac, the net transfers registered by gain of fluorescence (Dn → Ac) represent approximately (17% × 83%) = 14% of all transfers.

(ii) Initial rate (the signal increase from *t* = 5 − 15 s, after subtraction of background without LTP, as proportion of maximum signal) = (from Figure 5B in Watanabe 1.4%/s*et al.* [21]).

(iii) Since equilibrium requires 20 nM of LTP to transfer ∼200 nM of NBD–PA from Dn to Ac, equilibrium occurs when each LTP has undergone 200/20 ≈ 10 rounds of such transfer. Thus, the *in vitro* rate of traffic between any two liposomes is as follows:

$$\approx \frac{rate[=1.4\%]\;\times \;\text{number rounds}[=10]}{\text{fraction (transfers reported})\;[=14\%]}\approx 1\;/s$$

Additional analysis of transfer assays using radiolabelled PA in the place of NBD–PA from Connerth *et al.* [19] yields a similar rate, indicating that the NBD headgroup does not affect transfer. This rate is also comparable to that measured for other LTPs, for example, ORPs [31].

**(B) Estimate of Minimum *In Vivo* Rate of PA Import across the Mitochondrial Intermembrane Space in Budding Yeast**

This analysis of *in vivo* transfer, as shown in diagrammatic form in Figure 4B, uses three approximations to estimate the lower limit of transfer by Ups1 as ∼12/s: (i) the minimum synthesis rate of cardiolipin (CL) to reproduce its content in a single-cell cycle, (ii) the copy number of Ups1 per cell, and (iii) the proportion of PA import mediated by Ups1.

(i) The production of one CL requires delivery of two PA molecules to the inner membrane. Mitochondria in yeast have a similar surface area [88] and similar amount of lipid [89] to the plasma membrane, which has approximately 2 × 10^8^ phospholipids (PLs) [90]. Therefore, we assume that the number of mitochondrial PLs per cell is ∼2 × 10^8^. CL makes up ∼15% of mitochondrial PLs 91, 92, that is, 3 × 10^7^/cell. By comparison, levels of all precursors to CL are low (<10% of CL), and are discounted here. To make this amount of CL, the number of imported PA is 6 × 10^7^.

Mitochondrial CL must be made at least once per cell cycle (∼5000 s).

$$Import\;\;\;rate\;\;\;of\;\;\;PA\geq 6\times 10^{7}/5000=12000/s$$

(ii) The estimated copy number of Ups1 is ∼700 per cell [93]

(iii) Ups1 carries out approximately 70% of CL import (see Connerth *et al.* [19], Figure 1B).

$$\begin{array}{ll} Transfer\;\;by\;\;Upsl & \geq 0.7\times 12\;000/700\\ \; & \geq 12/s \end{array}$$

That is, ≥12-fold faster than rate measured *in vitro* – see the ‘Evaluation of *In Vitro* PA Transfer Assays by Ups1/Mdm35 (Yeast Homologs of PRELI/TRIAP1) in Watanabe *et al.* [21]’ section.

**Potential Sources of Error**

(i) Overestimate of Ups1 role: some experiments in Connerth *et al.* [19] indicate that *Δups1* has 50% CL synthesis of wild-type, not 30%, error factor × 1.4.

(ii) Overestimate of mitochondrial area; this might include an error by a further factor ×3.

If both (i) and (ii) apply, *in vivo* LTPs transfer rate is still ≥3/s that is, ∼3× faster than the measured rate.

Alt-text: Box 2

Almost any pair of organelles is linked by a contact site, meaning that lipid can traffic from A⇔B⇔C⇔A. In such a circular arrangement, blocking any one step still allows lipid to access all compartments. A well-explored example of this is the lipid traffic pathway ER ⇔ vacuole (yeast equivalent of lysosome) ⇔ mitochondria ⇔ ER. Blocking either of the routes involving mitochondria leads to hypertrophy of the other route, which is presumed to be a compensatory change that allows lipids to reach mitochondria 68, 69. Multiple circularities may explain how genetic screens fail to identify LTPs along key routes where lipid traffic is highly complex [70].

### What Is the Limit for Experiments in Living Cells?

A focus for the future will be to build up techniques that overcome the problems of studying some lipid transport pathways *in vivo*. Here we look at three techniques by which we might advance our understanding of the transfer of lipids between cellular compartments by LTPs.

#### Lipid Probes to Pulse and Chase

A major difficulty for *in vivo* studies of lipid traffic is to find a lipid reporter that allows the study of transport at high temporal and spatial resolution in an intact environment. The value of fluorescent lipids is often limited by the effects of large groups on the physicochemical properties of the native lipid. This can be partly overcome by using reporters with only minimal changes compared to the original lipid, such as additional conjugated double bonds in either cholesterol or acyl chains, although these molecules are hard to image 71, 72. Radiolabelled lipids or precursors have long been used to probe traffic pathways, and they continue to be useful 1, 73, although their value is limited for following intracellular location. One way to deliver a pulse of lipid is to use caged lipids. These are nonpolar precursors that are activated by light, and they can be highly informative [74]. Another way to pulse lipid into cells is to add a nonbilayer precursor, such as a lysolipid, that is rapidly converted into a bilayer lipid. For example, in yeast lyso-PS is acylated to PS in the ER within 1 min, allowing PS traffic out of the ER to be followed in a time-resolved manner 32, 34. In addition, lipids added externally can be minimally modified, creating so-called bifunctional lipids. Most often two small chemical groups are added, typically to the acyl chain: azide for photo-crosslinking and alkyne for detection (after fixation) by click chemistry [75]. So far, this pair of groups has been added only to lipid precursors such as fatty acid or sphingosine, but their use in bilayer lipids could be informative.

#### Protein Reporters

The localisation of a particular lipid can also be determined with a protein reporter that binds its headgroup. The reporter should be detectable when present at low stoichiometry, so its binding does not affect the total lipid pool. The reporter should also be nonbiased toward any given lipid pool, which has been achieved for PI4P detection using a bacterial reporter [76]. By contrast, detection of cholesterol with perfringolysin (subunit D) is biased for chemically active sterol not bound to other lipids, so a large proportion of sterol in the plasma membrane is undetectable [77]. Future advances might include the development of probes specific for PC, phosphatidylethanolamine, and PI, or that can identify all sterols.

#### Heterologous Replacement

In addition to directly studying lipid traffic, a proposed LTP activity can be inferred when its function is replaceable with an LTP with the same *in vitro* specificity but without sequence homology (hence heterologous). This has been demonstrated for StARD4, overexpression of which has similar functional consequences on sterol traffic as microinjection of cyclodextrin, a small sugar polymer that can transfer sterols [78]. Other experiments are less clear. Deletion of Ysp2p, a putative sterol transfer protein in yeast, is partially rescued by the unrelated sterol transfer domain from human StARD3 [73]. Similarly, deletion of Sec14, a PI/PC-specific member of the cellular retinaldehyde binding protein/triple functional domain protein (CRAL/TRIO) superfamily, is partially rescued by an unrelated PI/PC transfer protein from the StARkin superfamily [79]. The lack of strong rescue in both these cases may indicate a greater degree of complexity, for example, in intracellular targeting.

#### Genetics (Fast versus Slow Approaches)

Genetic manipulation of one or multiple LTPs has yielded many insights, but there are limits imposed by the ability of cells to adapt, in particular by hypertrophy of parallel pathways, as we have described. More subtle approaches may be developed to minimise cellular adaptations to deletion of LTPs. For example, the problems of cellular adaptation to loss of ER ⇔ mitochondrial LTPs 68, 80 can be mitigated by hyperactive Vps13p mutants that bypass that genetic lesion, hence preventing gross hypertrophy of vacuole ⇔ mitochondrial contacts 81, 82. Other approaches for the future include rapid relocation of LTPs within cells [83] or using small molecular inhibitors, such as the ORPhilins to inhibit ORPs [38].

## Concluding Remarks: The Eventual Goal Is to Align *In Vitro* and *In Vivo* Experiments

The function of some LTPs is still uncertain. Even though many LTPs are thought to mediate the net transfer of lipids along specific routes in cells, these routes have yet to be studied in detail. It would be a big advance if a combination of *in vitro* and *in vivo* techniques was applied to produce a coherent set of results for each route. One way to link *in vitro* and *in vivo* experiments, perhaps revealing the disparities between them, would be to focus (if only briefly) on estimates of the rate of lipid transfer arising from each approach. The extent to which these match each other indicates how close we are to a full understanding (see Outstanding Questions).

Taking sterol traffic in yeast as an example, it has been shown in yeast that Osh4 (30 000 copies per cell, the major ORP in numeric terms) can transfer sterol at a rate of up to 0.5/s [31]. It is therefore possible that it meets the demand for forward sterol traffic out of the ER to allow cellular replication (replication requires ∼10 000 sterols/s) [37]. However, this set of estimates must be reconciled with other studies that estimate the maximal rate of sterol traffic is tenfold higher [62]. Other LTP-mediated steps show discrepancies between rates of transfer measured *in vitro* and *in vivo*. We have used the literature to consider one example in detail: import of PA across the mitochondrial intermembrane space for cardiolipin synthesis by the Ups1/Mdm35 complex in yeast [19]. *In vitro,* the LTP complex can transfer PA at ≤1 lipid/s (Figure 4A and Box 2A) [21]. However, *in vivo*, we estimate each LTP complex imports ≥12 lipids/s (Figure 4B and Box 2B). Such a fast imputed rate for *in vivo* traffic is not unique. For example, StAR has been suggested to transfer sterol at 7/s [84]. In addition, fast nonvesicular lipid traffic is not limited to either sterols or import into mitochondria, as it appears to occur for ER to plasma membrane traffic of PS [34], though not all phospholipids have been tested. We interpret these findings not to say that the studies cited are in any way flawed. Instead, the discrepancies (here ≥12-fold) might stimulate further research.

To understand LTPs, lipid traffic *in vivo* should be measured accurately. Factors that might affect lipid transfer should be replicated for assays *in vitro*. Together, these steps may advance us toward a more united picture.

Outstanding Questions How many LTPs are there remaining to be identified, and what net transfer of lipids do they mediate? Increasing the sensitivity of bioinformatics searches may help.

What are the lipid specificities for different LTPs? What ligands are we missing, and which of the known ones are false? To what extent does *in vitro* data on LTP specificity match up to transport in cells? Do additional lipid ligands power countercurrents?

How often do LTPs not mediate net traffic of lipids? Developing specific assays for lipid sensing and lipid presenting roles for LTPs will more precisely dissect their overall functions.

What determines the donor and acceptor membrane specificities of LTPs with no accessory domains?

How does the occupancy of an LTP by a particular ligand affect its interaction with a specific membrane, and how often does this promote net traffic in cells? So far this has been found for Osh4 only *in vitro*, which deposits ergosterol extremely slowly to ER-type membranes.
